# Reduced serum BDNF levels are associated with the increased risk for developing MDD: a case–control study with or without antidepressant therapy

**DOI:** 10.1186/s13104-020-04952-3

**Published:** 2020-02-21

**Authors:** Md. Prova Zaman Emon, Rajesh Das, Nuruna Lovely Nishuty, M. M. A. Shalahuddin Qusar, Mohiuddin Ahmed Bhuiyan, Md. Rabiul Islam

**Affiliations:** 1 Department of Pharmacy, University of Asia Pacific, 74/A Green Road, Farmgate, Dhaka, 1215 Bangladesh; 2 Department of Psychiatry, Bangabandhu Sheikh Mujib Medical University, Dhaka, 1000 Bangladesh

**Keywords:** Serum BDNF, Major depressive disorder, MDD, Case–control study, Bangladesh

## Abstract

**Objective:**

We do not have any consistent markers for major depressive disorder (MDD) though various biological factors are involved in the pathophysiology. We aimed to evaluate the serum brain-derived neurotrophic factor (BDNF) levels in MDD patients with or without antidepressant therapy compared to healthy controls (HCs).

**Results:**

We assessed serum BDNF levels among three groups: drug-naïve MDD patients (n = 41), drug-treated MDD patients (n = 44), and age-and sex-matched HCs (n = 82). Serum BDNF levels were measured by enzyme-linked immunosorbent assay (ELISA) kit. Serum levels of BDNF were detected significantly lower in drug-naïve MDD patients compared to HCs. No significant alterations of serum BDNF levels between drug-treated patients and HCs were identified. Significant negative correlations between serum BDNF levels and Hamilton depression rating (Ham-D) scores were observed in both drug-naïve and drug-treated MDD patients. Receiver operating characteristic (ROC) analysis showed good diagnostic value for serum BDNF levels in drug-naïve MDD patients with the area under the curve at 0.821. The present study suggests that low serum BDNF levels may be involved in the pathophysiology of MDD. The reduced serum BDNF levels might be used as an early risk assessment marker for major depression.

## Introduction

Major depressive disorder (MDD) is a devastating psychiatric illness associated with a greater risk of disability that impairs quality of life [1]. MDD is the fourth leading cause of disability and the lifetime prevalence of MDD is 15% [2]. According to the World health organization (WHO), by 2020, MDD is assumed to be the second leading cause of global death [3]. The pathophysiology of MDD is very complex and even not completely understood. In most cases, the diagnosis depends on the structured questionnaire following the diagnostic and statistical manual (DSM). The treatment decision of MDD often complicates the physician because of its wide range of symptoms. Recent studies suggested the different genetic, environmental, biological, psychological, and social factors are involved in the pathophysiology and prognosis of depression [4–6].

Neurotrophic factors like nerve growth factor (NGF), brain-derived neurotrophic factor (BDNF) and glial cell line-derived neurotrophic factor (GDNF) are the critical signaling molecules in the neurodevelopment and maintenance of central and peripheral nervous systems [7]. These factors are responsible for the regulations of neurogenesis, neuronal growth, differentiation, and plasticity of neuronal networks, cell death, inflammation and autoimmune demyelination [8, 9]. Recently, the neurotrophic factors are extensively studied in MDD but the findings are not consistent. One neurotrophic hypothesis described that MDD is associated with disrupted neuroplasticity, and antidepressant possess the effect that can recover neuronal networks [10]. According to the monoamine hypothesis, MDD is associated with the inability of neuronal systems to exhibit appropriate adaptive plasticity and supported by the alteration of glial cell structure [11]. These theories suggested that glial cell volume in the cortical areas and limbic systems can be decreased in mood disorder which results in a change in the neuronal density and size [12].

Brain-derived neurotrophic factor is a member of the neurotrophin family of growth factors similar to other members of this family [13]. BDNF involves neurotransmitter modulator, learning, neuronal survival and proliferation, playing a critical role in growth, differentiation, maintenance, outgrowth, survival, repair, and synaptic plasticity of the neuronal system [14]. Several studies observed a significant association between serum BDNF levels and the development of MDD. Lowered serum BDNF levels were observed in MDD patients compared to healthy controls (HCs) [15, 16]. One study reported higher serum BDNF levels in MDD patients compared to HCs [17]. Another study found no significant alterations of serum BDNF levels between MDD patients and HCs [18]. Some studies reported no significant difference in serum BDNF levels before or after antidepressant therapy [19, 20]. Moreover, long-term antidepressant therapy stimulates the expression of BDNF and neurogenesis in the animal model [21]. Thus the present study aimed to evaluate the serum levels of BDNF in MDD patients with or without antidepressant treatment among the Bangladeshi population.

## Main text

### Methods and materials

#### Study population

This study enrolled 41 drug-naïve MDD patients, 44 drug-treated MDD patients, and 82 age-and sex-matched HCs who met the criteria of DSM, 5th edition (DSM-5). All patients were recruited from Bangabandhu Sheikh Mujib Medical University (BSMMU), Dhaka, Bangladesh with a history of MDD symptoms for at least 2 weeks whereas HCs were from different parts of Dhaka city. A structured questionnaire was applied for the documentation of socio-demographic profiles of the study population. The severity of depression was measured by the Ham-D scale and participants with Ham-D scores greater than 7 were considered cases. Exclusion criteria included the history of other psychiatric disorders like delusions, mental retardation, bipolar disorder, schizophrenia, personality disorder, mood-congruent or incongruent psychotic features, comorbid psychiatric illness, neurological disease or patients with clinical evidence of dementia. Volunteers who met the above exclusion criteria and not diagnosed as MDD according to DSM-5 by a qualified psychiatrist were taken as HCs. Ham-D scores of HCs were also below 7. Patients having severe or acute medical illnesses, presence of infectious disease, immune disorders, abnormal body mass index (BMI), alcohol or narcotic drug dependency also excluded from this study.

#### Blood sample collection and serum separation

A 5 mL blood samples were collected from the cephalic vein of each participant in the morning between 8.00 AM to 9.00 AM after an overnight fast. Collected blood samples were allowed to clot for 1 h without any shaking or agitation in a fixed place at room temperature. The clotted samples were then centrifuged at 1000×*g* for 15 min. The clear aliquots were carefully separated and stored at − 80 °C until further analysis.

#### Measurement of serum BDNF levels

Serum BDNF levels were quantified by commercially available enzyme-linked immunosorbent assay (ELISA) kit (BosterBio, USA) according to the manufacturer’s instructions. The sensitivity or the minimum detectable value was < 15 pg/mL. Intra-assay and inter-assay coefficient of variation (CV) was 4.6% and 7.3%, respectively. The average recovery rate was 91.3% for this assay. There was no cross-reactivity with other neurotrophic factors present in the serum sample. The analysis was carried out by the same person for cases and controls who were unaware of the outcome of the study.

#### Statistical analysis

The Mann–Whitney U test was used for continuous variables and Fisher’s exact test for categorical variables. Data were presented as mean ± standard error mean. Spearman’s rank correlation test was applied to find the correlation between serum BDNF levels and the severity of depression. Variations among data were visually demonstrated by bar diagrams. Receiver operating characteristic (ROC) analysis was performed to evaluate the diagnostic performance of serum BDNF levels. Results were considered significant where P values were less than 0.05. All the statistical analyses were performed by the statistical package for social sciences (SPSS) version 23.0 (IBM Corp., Armonk, NY).

### Results

**Fig. 1:**
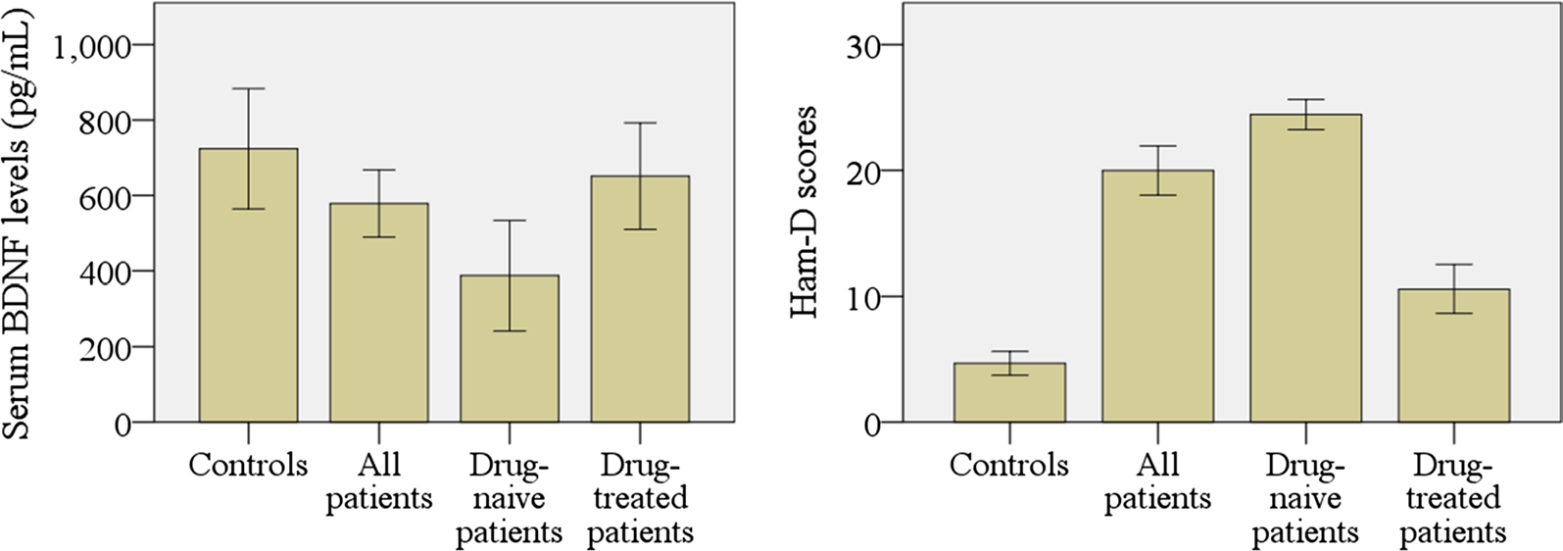
Comparison of serum brain-derived neurotrophic factor (BDNF) levels and Hamilton depression rating (Ham-D) scores among the different study populations. A significant difference between patient and control groups at a 95% confidence interval

**Fig. 2:**
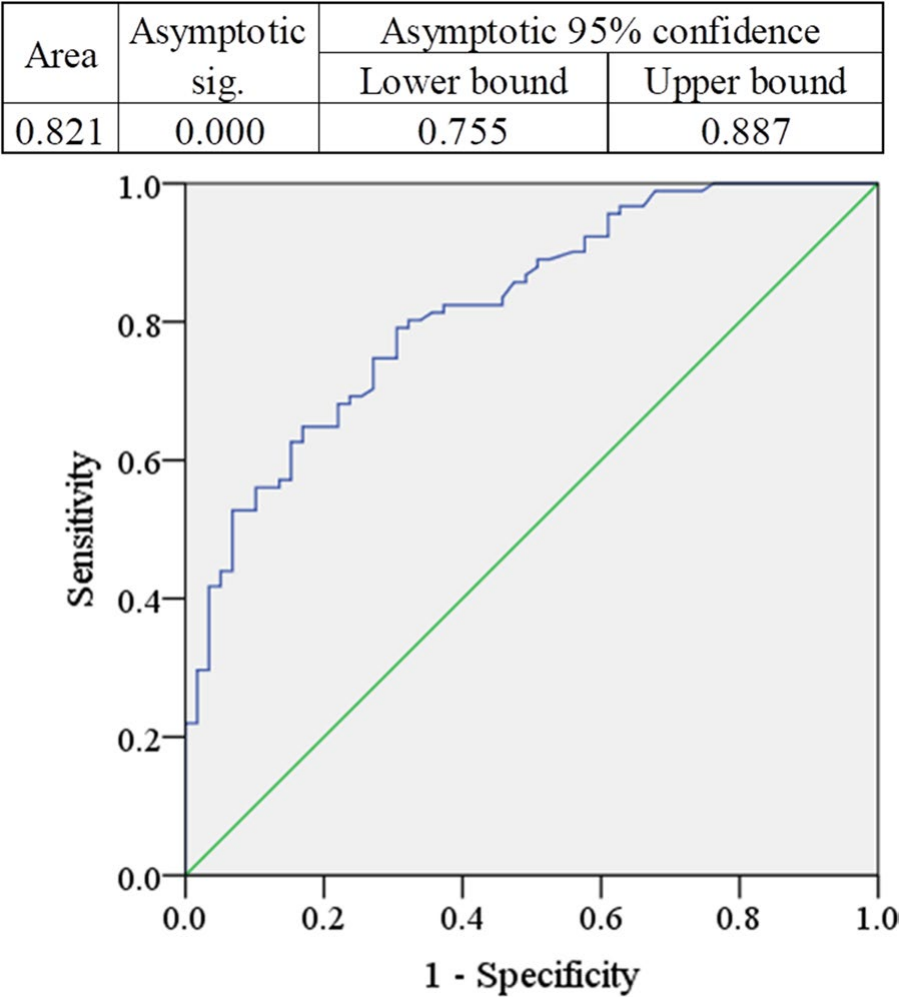
Receiver operating characteristic (ROC) curve serum brain-derived neurotrophic factor (BDNF) in drug-naïve MDD patients. The cut-off point was detected as 221.05 pg/mL

The study groups were similar in terms of age, gender, and BMI (Table 1). A significant decrease in serum BDNF levels was observed in drug-naïve MDD patients compared with the HCs (P < 0.05). On the other hand, an elevation of serum BDNF levels was detected in antidepressant treated MDD patients when compared with HCs. Among 44 drug-treated MDD patients, sertraline (dose 50–100 mg/day), paroxetine (dose 20–40 mg/day), venlafaxine (dose 75–100 mg/day), escitalopram (dose 10 mg/day), and fluoxetine (dose 40–60 mg/day) were prescribed for 16, 12, 10, 4, and 2 patients, respectively. Spearman’s correlation study showed a significant negative correlation between serum levels of BDNF and Ham-D scores in drug-naïve MDD patients (r = 0.797; P < 0.001) and antidepressant treated MDD patients (r = 0.758; P < 0.001). Changes of serum BDNF levels among the study population were presented by the bar diagram (Fig. 1). Moreover, ROC analysis revealed a good diagnostic value for serum BDNF levels in drug-naïve MDD patients (Fig. 2).

**Table 1:** The characteristics, clinical features, and laboratory findings of the study population

Parameter	Controls (n = 82)	All patients (n = 85)	Drug-naive patients (n = 41)	Drug-treated patients (n = 44)	p^a^ value	p^b^ value	p^c^ value	p^d^ value
Age in years	31.13 ± 1.82	33.37 ± 1.10	34.13 ± 1.50	32.64 ± 1.63	0.300	0.207	0.545	0.504
Gender (F/M)	50/32	48/37	26/15	31/13	0.572	0.732	0.851	0.635
BMI (kg/m^2^)	24.83 ± 0.75	26.60 ± 0.65	25.18 ± 0.90	27.97 ± 0.87	0.137	0.779	0.112	0.142
Ham-D score	4.96 ± 0.55	19.96 ± 0.29	23.90 ± 0.42	11.01 ± 0.43	0.000*	0.000*	0.000*	0.008*
Serum BDNF (pg/mL)	723.77 ± 78.36	578.62 ± 44.65	387.80 ± 70.44	651.21 ± 69.24	0.083	0.002*	0.105	0.009*

Age, BMI, Ham-D score, and the value of BDNF are shown in mean ± SEM. F/M, female/male; BMI, body mass index; Ham-D, 17-item Hamilton depression rating scale; p^a^, comparisons between controls and all patients; p^b^, comparisons between controls and non-treated patients; p^c^, comparisons between controls and treated patients; p^d^, comparisons between non-treated and treated patients

*P < 0.05 (Significant difference between patient and control groups at 95% confidence interval)

### Discussion

According to the present study, decreased serum BDNF levels were observed in drug-naïve MDD patients compared to HCs whereas no significant changes were found when compared between drug-treated MDD patients and HCs. We found a significant negative correlation between the serum BDNF levels with the severity of depression in both drug-treated and non-treated MDD patients. Our results can be described by different hypotheses. One study stated that lower levels of neurotrophic factors have a significant role in the development of depression whereas the increase in serum levels occurs after antidepressant therapy [15]. Another hypothesis is based on BDNF response to different stress mechanisms. Stress may reduce serum BDNF levels which can cause neuronal damage, neuronal atrophy and death in the hippocampus region of the brain result in biological vulnerability [22]. Treatment of MDD patients by antidepressants can increase both the BDNF level and neurogenesis [21]. Karege et al. [23] reported the serum/blood BDNF ratio was lower in MDD compared to HCs and this altered levels of serum BDNF may interfere with the BDNF release mechanisms. Studies proposed that plasma serotonin level is negatively correlated with the severity of depression and decreased levels of serotonin is released in MDD patients [24]. Another research suggested that BDNF in plasma is mainly stored in platelets and their release is correlated with each other, as a result, there is a correlation between serum BDNF levels with serotonin and depression [23, 24].

The present study results are consistent with the many previous studies. Karege et al. observed serum BDNF levels were significantly lower in MDD patients compared to HCs. Also, the levels were negatively correlated to the severity scores and the female patients were high severity scores and low BDNF levels than men [15, 23]. Bocchio-Chiavetto et al. [25] found significantly decreased serum BDNF levels, but not plasma, in MDD patients than those of HCs. Another study found that plasma BDNF levels were significantly lower in MDD patients than in the HCs [26]. Ozan et al. [27] found MDD patients had a lower serum BDNF level than HCs and male subjects had a higher serum BDNF level than female subjects. Yoshida et al. [28] reported that serum levels of mature BDNF, but not proBDNF, were significantly lower in MDD patients than those of HCs. Another recent study revealed that serum BDNF levels were significantly reduced in MDD patients compared to HCs [29]. Gupta et al. [30] stated that after the treatment of MDD patients with fluoxetine and agomelatine, the serum BDNF levels were increased. Serum BDNF levels were significantly decreased compared to HCs in MDD patients with bipolar symptoms [31]. Moreover, one study found that serum BDNF was significantly decreased in the drug-naive MDD patients than in the antidepressant treated MDD patients or in the HCs [32]. Additionally, many studies reported that the elevation of serum BDNF levels was observed after antidepressant treatment in MDD patients compared to HCs. One recent study suggested that low serum BDNF levels were associated with MDD and after the treatment with escitalopram positive effect was found [33]. According to Matrisciano et al. [34] serum BDNF levels in MDD patients were lower than HCs and after 5 weeks of treatment with sertraline, serum BDNF levels were increased significantly. Lener et al. [35] predicted that the antidepressant action of ketamine increases the serum levels of BDNF in depression.

Similar to depression, some researchers observed an association of serum BDNF levels with anxiety and stress. One study found decreased serum BDNF levels in post-traumatic stress disorder after trauma compared to HCs [36]. Moreover, some other serum neuropeptides like GDNF, vascular endothelial growth factor, NGF, insulin-like growth factor-I, etc. can also be a pathological identifier of MDD. Diniz et al. [37] found decreased serum GDNF levels in MDD patients compared to HCs. Another study reported MDD patients were detected with significantly lower serum GDNF levels than HCs [38]. A post-mortem and imaging study also reported lower serum levels of GDNF in patients suffering MDD compared to HCs [39]. Also, reduced serum NGF levels were detected in depressed patients compared to HCs [40].

Another important finding of the present study is the ROC-curve analysis which deals with the diagnostic or predictive value of the analyzed parameters. Many past studies measured the serum BDNF levels in MDD patients and identified its association with depression but none of them were evaluated for their accuracy in diagnostic or predictive purpose [15, 31]. In the present study, the ROC-curve was plotted for serum BDNF levels to assess the diagnostic or predictive value in drug-naive MDD patients. The area under the curve for ROC analysis was 0.821 and the cut-off point was detected as 221.05 pg/mL. Higher values were allotted as the disease condition. Sensitivity, specificity, positive predictive value and negative predictive value were 72.4%, 73.1%, 68.3%, and 78.5%, respectively.

The finding of decreased serum BDNF levels in drug-naïve MDD patients may indicate a significant relationship between the pathophysiology of depression and peripheral levels of BDNF. The negative correlation between Ham-D scores and serum BDNF levels in MDD patients can be used as a predictor for the severity of depression. Increased serum BDNF levels in antidepressants treated MDD patients supported the drug-induced expression of peripheral BDNF. Moreover, analyzed serum BDNF levels achieved good diagnostic value for drug-naive MDD patients which can be used as predictors for the assessment of depression risk. The positive aspect of the present study is that we have firmly controlled age, sex, BMI, and other socio-demographic profiles between the groups. Exclusion criteria were equally followed for both cases and controls. We tried to maintain a more homogenous study population to get precise results.

## Limitation of the study

To the best of our knowledge, this is the first study regarding the evaluation of serum BDNF levels in MDD patients among the Bangladeshi population though it has few limitations. We didn’t perform pre and post measurement of serum BDNF levels in the same patient set for antidepressant therapy as well as any food intake data was not recorded. These outcomes should be treated as preliminary and further studies with a large and more homogeneous population are required to understand the exact relation between serum BDNF levels and major depression.

## Data Availability

Data supporting our findings are available from the corresponding author on reasonable request.

## Abbreviations

BDNF: Brain-derived neurotrophic factor
BMI: Body mass index
BSMMU: Bangabandhu Sheikh Mujib Medical University
DSM: Diagnostic and statistical manual
GDNF: Glial cell line-derived neurotrophic factor
Ham-D: Hamilton depression rating scale
HC: Healthy control
MDD: Major depressive disorder
NGF: Nerve growth factor
ROC: Receiver operating characteristic
